# Seeking a Mechanism for the Toxicity of Oligomeric α-Synuclein

**DOI:** 10.3390/biom5020282

**Published:** 2015-03-25

**Authors:** Hazel L. Roberts, David R. Brown

**Affiliations:** Department of Biology and Biochemistry, University of Bath, Claverton Down, Bath BA2 7AY, UK; E-Mail: H.L.Roberts@bath.ac.uk

**Keywords:** α-synuclein, neurodegeneration, Parkinson’s disease, aggregation, toxic oligomers, amyloid fibrils

## Abstract

In a number of neurological diseases including Parkinson’s disease (PD), α‑synuclein is aberrantly folded, forming abnormal oligomers, and amyloid fibrils within nerve cells. Strong evidence exists for the toxicity of increased production and aggregation of α-synuclein *in vivo*. The toxicity of α-synuclein is popularly attributed to the formation of “toxic oligomers”: a heterogenous and poorly characterized group of conformers that may share common molecular features. This review presents the available evidence on the properties of α-synuclein oligomers and the potential molecular mechanisms of their cellular disruption. Toxic α-synuclein oligomers may impact cells in a number of ways, including the disruption of membranes, mitochondrial depolarization, cytoskeleton changes, impairment of protein clearance pathways, and enhanced oxidative stress. We also examine the relationship between α-synuclein toxic oligomers and amyloid fibrils, in the light of recent studies that paint a more complex picture of α-synuclein toxicity. Finally, methods of studying and manipulating oligomers within cells are described.

## 1. Introduction

α-synuclein is a 14 kDa intrinsically-disordered protein with strong links to neurodegenerative disease. Oligomeric forms of α-synuclein are thought to be responsible for the neurotoxic effects of α-synuclein; a popularly supported concept known as the “toxic oligomer hypothesis” [1]. Not all α-synuclein oligomers are toxic, and there is no definition for a “toxic oligomer”, other than its detrimental effect to the viability of cultured cells. It is thought that a common conformation exists between the toxic oligomers of α-synuclein and other aggregate-forming proteins, such as amyloid-β, and that this involves a high amount of β-sheet secondary structure [2]. We also have a crude idea of which cellular functions are affected by α-synuclein toxic oligomers. However the information is patchy at best, and conflicting at worst; perhaps due to the difficulties of studying a dynamic, heterogenous population of oligomeric species. A better understanding of the structure and cellular effects of toxic oligomers would be highly beneficial to focusing drug discovery efforts.

The last few years have brought new models and techniques to the study of α-synuclein oligomer toxicity. Questions have also been raised about whether the “toxic oligomer hypothesis” is an oversimplification of the *status quo*. This review will firstly introduce the neurodegenerative diseases that are known to involve α-synuclein aggregation. It will then address the nature of α-synuclein monomers, and individually discuss small oligomers, larger proteinase-K resistant oligomers, and amyloid fibrils. Evidence for toxicity and disruption of different cellular functions will be appraised for each group of α-synuclein species. Finally, new theories on the relationship between oligomers and fibrils in cellular toxicity will be presented, and the methodologies used to study oligomer toxicity briefly outlined.

## 2. Pathology of Synucleinopathies

Synucleinopathies are a group of neurodegenerative diseases where Lewy bodies and Lewy neurites, fibrillar cytoplasmic inclusions composed largely of α-synuclein, are found within neuronal cells [3]. These include Parkinson’s disease (PD), Parkinson’s disease dementia (PDD), and Dementia with Lewy bodies (DLB). In these diseases, α-synuclein pathology in the *substantia nigra* (SN) is closely correlated with motor symptoms and the death of SN dopaminergic neurons innervating the striatum. Dementia in PDD and DLB is associated with Lewy bodies occurring in the cortex, potentially having spread by cell-to-cell transmission from limbic regions [4]. Multiple System Atrophy (MSA) is another synucleinopathy, but is distinct as oligodendroglia are the bearers of α-synuclein-positive inclusions, rather than neurons [5].

Although not primarily implicated in the cause of Alzheimer’s disease (AD), fragments of α-synuclein are also found in extracellular “amyloid plaques” of AD brains. Additionally Lewy bodies occur in 32%–57% of sporadic AD [6,7,8]. Even in the absence of visible α-synuclein aggregates, levels of soluble α-synuclein in the cortex of AD brains are often double the level of controls, and strongly correlate with cognitive impairment [9].

**Figure 1:**
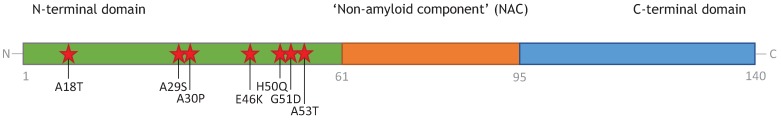
Location of disease-associated point mutations in α-synuclein, indicated by red stars.

Interest in the toxicity of α-synuclein began when mutations of the SNCA gene encoding the protein were identified in cases of familial PD, and later duplications and triplications of the gene were associated with familial and sporadic PD [10,11]. Missense mutants appear to have an earlier age-of-onset than sporadic cases of PD, and faster rate of motor decline [12]. All of the missense mutations identified to date are notable for being confined to two helix-forming regions of the N-terminal domain [12], and include: A30P [13], E46K [14], A53T [15], H50Q [16], and G51D [17]. Additionally, two more were recently discovered that potentially add new phosphorylation sites to the first N-terminal helix [18]. Figure 1 illustrates the location of disease-associated point mutations in α-synuclein. The toxicity of these α-synuclein variants appears to stem from their enhanced aggregation into oligomers and amyloid fibrils [1,19]. Single-molecule force spectroscopy of A30P, E46K, and A53T α-synuclein has highlighted their destabilizing effect on the N-terminal domain and increased propensity for forming β-structure, which may promote aggregation [20]. *In vitro* A30P appears to differ from A53T and E46K in that it forms fibrils more slowly than the wildtype, although readily aggregating into soluble protofibrillar oligomers [21]. However, there is no evidence of inhibited fibrillization *in vivo*; humans with the A30P variant have extensive Lewy bodies upon autopsy [5].

Increased expression of wildtype α-synuclein also increases formation of α-synuclein oligomers and fibrils, likely due to levels of α-synuclein exceeding the ability of cells to maintain proteostasis by chaperoning or degradation [5]. Some sporadic cases of PD may originate from increased expression of α-synuclein. GWAS studies have correlated polymorphisms in the SNCA gene with increased risk to PD, including the “Rep1” variant in the SNCA promoter [22]. Epigenetic changes can also enhance SNCA expression, including reduced CpG methylation of the SNCA intron 1, discovered in sporadic PD brains [23].

Sporadic PD may additionally result from a number of age-related changes to the brain that gradually tips the balance in favour of α-synuclein aggregation. These include chronic neuroinflammation, oxidative insults, changes to metal homeostasis, or a reduction in the efficiency of protein degradation pathways or chaperone production [24]. Recently transglutaminase 2, a protein cross-linking enzyme that has enhanced expression in PD patients, has also been shown *in vivo* to promote α-synuclein aggregation and toxicity [25]. The factors that lead to enhanced aggregation of α-synuclein are beyond the scope of this article, but have been reviewed elsewhere [5].

## 3. Monomeric α-Synuclein

In the cell α-synuclein is primarily monomeric and cytosolic [5,26], existing in a disordered state. Although the monomer has high conformational flexibility, it is more compact than a random-coil polypeptide of the same length. The protein rapidly fluctuates between an ensemble of preferred conformational states that are stabilized by transient long-range contacts, which form between the central 30–100 residues and the C-terminal 120–140 residues. In part, the contacts are electrostatic, as the C-terminus has a strong negative charge and the central region is weakly basic, and additionally contacts involve the burial of hydrophobic residues [27].

Up to a third of the cellular α-synuclein population is estimated to be bound to synaptic membranes [28]. Upon binding membranes, the N-terminal and central domains of α-synuclein fold into two amphipathic α-helices, whereas the acidic C-terminal 101–140 residues remain unstructured [29]. α-Synuclein has a preference for lipids with acidic headgroups and membranes with high curvature, such as small synaptic vesicles [30]. Localization to vesicles within the presynaptic nerve terminal is potentially important for its main physiological function, but a precise role has not been defined. A prevailing hypothesis is that α-synuclein chaperones the formation of SNARE complexes for vesicle fusion [31], perhaps through its direct interaction with the v-SNARE synaptobrevin 2 [32]. A recent study indicates that α-synuclein may only enhance SNARE complex assembly after oligomerizing on the membrane into an ordered α-helical array, of eight or more units [33]. Thus oligomers may be important for α-synuclein function, as well as dysfunction, with different folding pathways implicated for each.

There is very little evidence of a pathological role for the monomer alone. Inferences of monomer toxicity must be treated with caution, due to the ease at which α-synuclein interconverts dynamically between monomers and oligomeric species. *In vitro* assays for membrane permeabilization have indicated that recombinant monomers can disrupt membranes, although more weakly than the oligomers tested [29]. This could be interpreted two ways: either monomers in a high enough concentration are sufficient to deform membranes of anionic large unilamellar vesicles [34,35], or their tendency to spontaneously oligomerize upon membrane-binding is responsible [33]. Membrane disruption by oligomers will be discussed in Section 4. Another way that monomeric α-synuclein might plausibly exert toxicity is via interactions with copper and iron. Monomers, and even N-terminal peptides, may enhance the copper-catalyzed production of hydrogen peroxide *in vitro*. Excess reactive oxygen species (ROS) are speculated to be responsible for the depletion of reduced glutathione in the *substantia nigra* of PD brains [36]. Finally, there is evidence that monomeric α-synuclein has the ability to activate TLR4 receptors on microglia and astroglia, resulting in pro-inflammatory activation [37]. This activation is enhanced by A30P and E46K disease-associated mutations [38]. Activation of microglia and astroglia leads to chronic neuroinflammation in PD and other α-synucleinopathies, and may contribute to the degeneration of dopaminergic neurons [37].

## 4. Oligomers of α-Synuclein and Their Toxicity

### 4.1. Dimers, Trimers, and Tetramers

Dimers of α-synuclein are considered to be unstable and transient, although covalently cross-linked dimers and trimers have been generated *in vitro* under conditions of oxidative or nitrosative stress [39]. A recent study of α-synuclein dimers using single molecule AFM shows that they have several discrete structural conformations. Fibril-promoting A53T and E46K mutations increased the propensity of central and C-terminal segments to form β-structure, and increased formation of multiple intermolecular contacts in the dimer. In contrast, the A30P mutant protein, which is predisposed to form oligomers over fibrils *in vitro* [21], tended to have single points of intermolecular contact in the dimer [40]. Clearly dimers, like monomers, can contribute to a number of different pathways of oligomerization.

Similarly, trimeric α-synuclein is rarely studied as it only exists in mixed populations with monomers and tetramers. However, one study flagged up this mixed population of “small oligomers” for potentially influencing mitochondrial dynamics. Trimers, in a mixture with monomers and a few tetramers, were responsible for driving liposome clustering *in vitro*; an artificial model of mitochondrial fragmentation. In this model, monomers alone, fibrils or “mature oligomers” (both generated by 60 h of stirring) had no appreciable effect on the liposomes. Mitochondrial fragmentation predicts cell death in culture, but both factors increase dose-dependently with α-synuclein expression levels [41]. Thus it remains unclear whether this is a cause of α-synuclein toxicity.

Metastable α-helical tetramers, reported in 2012, have proved to be highly controversial, with a flurry of publications subsequently made in favour [42,43,44] or against [5,26] their existence. Up to 70% of native cytosolic α-synuclein could be in this form [44]. The tetramer is hypothesized to be a non-toxic species that inhibits fibril-forming pathways, and is disrupted *in silico* by disease-associated point mutations [45]. However, attempts to independently verify the existence of tetramers have had mixed success, so this remains a polarizing issue in the field [5].

### 4.2. Soluble β-Rich Oligomers

Despite there being little consensus on the mechanisms of α-synuclein toxicity, soluble oligomers rich in β-structure are widely regarded to be the main culprit. α-Synuclein is capable of forming native membrane-associated oligomers with α-helical internal structure, but stable oligomers implicated in toxicity studies are invariably β-rich. Soluble oligomers of α-synuclein are heterogeneous, but for smaller oligomers the extent of internal β-sheet structure is believed to be more important to predicting toxicity than size [46]. β-sheet structure can be measured indirectly for isolated populations of oligomers using a variety of techniques including proteinase-K resistance, circular dichroism, ATR-FTIR spectroscopy, binding of the fluorophore 1-anilino-naphthalene-8-sulfonate (ANS) or conformational antibodies, such as A11 or FILA-1 [2,47,48,49,50,51].

The conditions of oligomerization are important for their resulting conformation. Oligomerization *in vitro* can be promoted by agitation (stirring or shaking) either in physiological buffer alone [47,52], or with the inclusion of 5%–30% ethanol, Fe^2+^, Cu^2+^, or dopamine to aid misfolding [53,54,55,56]. These methods all produce toxic soluble oligomers, but there are reasons to believe that they are not similar in structure or cellular effects. Groups using agitation in physiological buffer report spherical or disc-shaped oligomers that associate with membrane bilayers and induce membrane permeabilization [47]. However, dopamine-bound α-synuclein oligomers are short rod-shaped structures that do not bind phospholipids or affect membrane permeability [57], but may cause loss-of-function effects with respect to SNARE-complex chaperoning [55]. Copper-binding also reduces the membrane association of α-synuclein, and induces the formation of distinctive soluble stellate oligomers [58,59]. If these species all exist physiologically, then clearly there is more than one variety of toxic α-synuclein oligomer.

**Figure 2:**
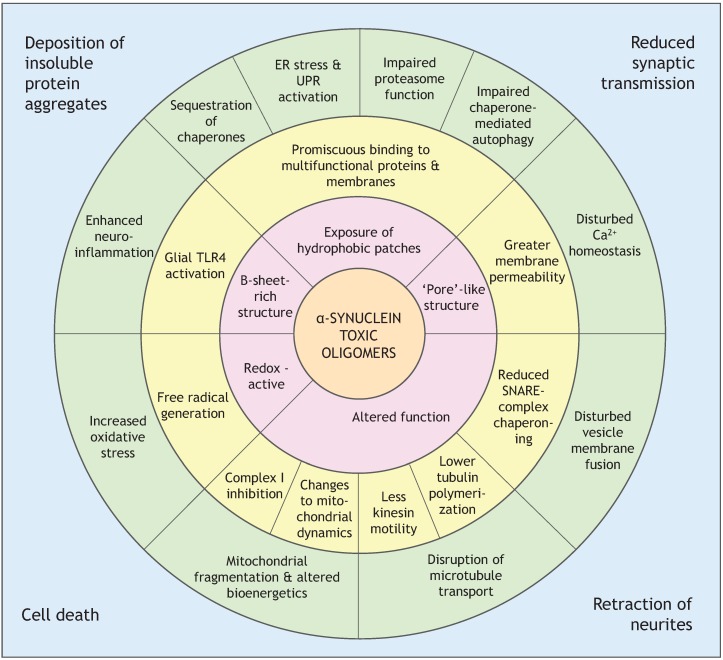
Cellular effects of α-synuclein toxic oligomers, and potential links to oligomer properties. *Inner shell*: proposed properties of toxic oligomers. *Middle shell*: examples of molecular effects conferred by toxic oligomers. *Outer shell*: cellular systems disrupted by toxic oligomers. *Edges of the box*: pathological outcomes of neuron dysfunction. ER—endoplasmic reticulum. UPR—unfolded protein response.

An important question that still remains to be answered is: what are the molecular features of a toxic oligomer that confer toxicity? Research on other amyloidogenic proteins and their toxic oligomers provides some clues. Toxic and non-toxic oligomers of similar size and shape were compared for the bacterial protein HypF-N, and one of the major differences between them was their ability to bind ANS. ANS is a dye that binds tightly and non-specifically to hydrophobic patches on proteins, and revealed in this case that toxic oligomers expose more hydrophobic residues. Toxic oligomers also had a less well-packed hydrophobic core and greater structural flexibility, which allowed them to insert into membranes and create Ca^2+^ permeability. Non-toxic oligomers only associated loosely with membranes, and did not permeabilize them [60]. The enhanced exposure of hydrophobic surfaces could additionally mediate aberrant binding to multifunctional proteins, such as the 26S proteasome complex, or increase the formation of ROS [50,61,62]. Figure 2, at the end of this section, shows the hypothesized links between features of α-synuclein toxic oligomers and cellular effects. Evidence supporting various mechanisms of cellular toxicity by α-synuclein oligomers are discussed henceforth.

#### 4.2.1. Membrane Permeability

Oligomeric species of α-synuclein have been shown to permeabilize lipid bilayers to calcium and other cations. Hexamers, pentamers, or octamers have been suggested to form on membranes in annular pore-like rings, based on electron microscopy and molecular simulations [48,56,63,64]. However, larger α-synuclein oligomers have also been characterized by a number of groups, and shown to have potent membrane-permeabilizing properties *in vitro* [47,52,53,54]. Lorenzen and colleagues characterized a globular ellipsoid containing 30 ± 6 monomers, with both a compact interior of antiparallel β-sheets, and a surrounding shell of disordered peptides that makes up 50% of the mass [47]. Membrane permeability is frequently modelled *in vitro* by measuring release of the fluorescent dye calcein from filled artificial vesicles. The study by Lorenzen *et al.* found all fractions of α-synuclein applied to anionic DOPG vesicles were able to induce some calcein release. However the 30 meric oligomer was estimated to be 17 times more potent, by concentration, than monomers; five times more potent than fibrils; and twice the potency of larger oligomers [47].

The mechanism by which membranes are made more permeable to small non-specific cations is contentious. One popular theory is the “pore-forming” hypothesis, where oligomers form a membrane-spanning pore, which may be shaped as a β-barrel similar to α-hemolysin. This is reviewed in some detail in [65]. To summarize, this hypothesis is supported by cryo-electron microscopy of annular oligomers in membranes [56], single-channel electrophysiology that appears to show discrete stepwise changes consistent with pore opening and closing [66], and evidence that oligomer-induced permeability is inhibited by both anti-aggregation compounds [66] and the oligomer-specific A11 antibody [67]. Interestingly the A11 conformational antibody also binds to pore-forming proteins α-hemolysin and perforin, and blocks their channel activity, allowing direct comparisons to be drawn with the conformation of α-synuclein [67].

Some researchers are not convinced of the existence of α-synuclein pores. Membrane permeability can be induced by a heterogenous range of α-synuclein oligomers and fibrils, which does not support a well-defined pore-like species being solely responsible. Membrane permeability induced by α-synuclein oligomers is also highly sensitive to lipid bilayer charge and packing, which seems more compatible with transient association of α-synuclein with the membrane, rather than the relatively stable insertion of pores [47,52]. Some electrophysiological studies also have failed to see discrete stepwise changes in conductance that would indicate oligomer-pore opening and closing [68,69]. On the other hand, this result is dismissed by supporters of the “pore hypothesis” as an artifact [65]. A study of Aβ oligomers highlighted that discrete changes in conductance can be masked by residual hexafluoroisopropanol used in oligomer preparation, but are restored by purging samples with nitrogen [70]. Nevertheless, theoretically α-synuclein oligomers could increase membrane permeability without forming physical channels through the membrane. Insertion of many hydrophobic resides by α-synuclein into a localized area of the lipid bilayer may disrupt lipid packing, and cause “thinning” of the membrane [71]. Additionally enhanced lipid flip-flop in the presence of α-synuclein oligomers has been observed [72]. The effectiveness of the membrane as a hydrophobic barrier is likely to be compromised as a result [71].

#### 4.2.2. Mitochondrial Dysfunction

Mitochondrial dysfunction is potentially a central feature of PD, as implied by the PD-like effects of mitochondrial toxins *in vivo*, and the ability of mutated mitochondrial proteins to cause familial PD. However, the connection between α-synuclein and mitochondrial dysfunction is poorly understood, as reviewed in [73]. α-Synuclein has been detected in the mitochondria of PD brain samples and associated with decreased complex I activity [74]. α-Synuclein was immuno-precipitated with complex I, but the participation of soluble oligomers was not studied [74]. Other research groups have not consistently reported the presence of β-rich oligomers in mitochondria, or the direct interaction of α-synuclein with complex I [75].

Few investigations have been made into the specific impact of α-synuclein soluble oligomers on mitochondria. It has also been suggested that α-synuclein toxic species could increase mitochondrial membrane permeability, based on the anionic cardiolipin-rich properties of the membrane [52], but a recent study failed to detect this [76]. Isolated mitochondria, incubated with soluble prefibrillar oligomers of α-synuclein, showed increased sensitivity to Ca^2+^-induced opening of the “mitochondrial permeability transition pore” (mPTP). Fibrils and monomers had no effect. However, Ca^2+^ entry could be entirely blocked by inhibiting the mitochondrial Ca^2+^ uniporter, so the oligomers were not inducing membrane leakage of Ca^2+^. Based on kinetics, the authors surmised that α-synuclein oligomers influenced recruitment and assembly of mPTP components in the “lag phase” [76].

Indirect effects of α-synuclein oligomers on mitochondria are perhaps more likely. These may include: increased fragmentation of mitochondrial networks, increased turnover through mitophagy, and increased association of ER with mitochondria [41,73]. Additionally, mitochondrial health may be disturbed by α-synuclein oligomers compromising other aspects of cell function. For example, mitochondrial energy status is strongly inter-linked with cytoskeletal health; stabilizing microtubule networks pharmacologically has been shown to improve mitochondrial function [77].

#### 4.2.3. Altered Cytoskeleton Formation

α-Synuclein oligomers may also be involved in changes to cytoskeletal integrity. Reduced tubulin polymerization has been detected in mouse dopaminergic neurons, where recombinant soluble oligomers were applied (roughly 160 kDa in size [78]). In this study no *in vitro* effect on tubulin polymerization was apparent [79], but another *in vitro* system revealed that soluble α-synuclein oligomers may inhibit the microtubule-polymerizing activity of the tau protein, and additionally impair kinesin motility [80]. Reduced tubulin polymerization might also be linked to a cell’s energy status. Diminished complex I activity was measured in mouse dopaminergic neurons treated with α-synuclein oligomers. Interestingly, overexpressing the neuroprotective DJ-1 protein in cells prevented the effects of oligomeric α-synuclein upon mitochondrial function and tubulin polymerization, and enhanced cell viability [79].

#### 4.2.4. Enhanced Formation of Reactive Oxygen Species (ROS) and Neuroinflammation

The enhanced accumulation of reactive oxygen species (ROS) is linked to α-synuclein expression [81], which may in part be a consequence of α-synuclein oligomers disturbing mitochondrial respiration, and uncoupling oxidative phosphorylation [73]. Mitochondria-independent increases in ROS have also been observed in rat primary neuronal cultures applied with α-synuclein oligomers. In this study proteinase-K resistant oligomers were more potent than proteinase-K sensitive oligomers, and fibrils and monomers had no significant effect [82]. Cytosolic ROS generation in response to α-synuclein oligomers may partly originate from NADPH oxidase, a superoxide-generating enzyme; its expression in neurons is normally low but upregulated during conditions of stress [46,82]. A direct contribution from α-synuclein is also possible. Copper bound to α-synuclein oligomers can catalyze ROS formation, and has been shown to enhance oligomer toxicity in cells and *in vitro* [36,58,59].

*In vivo*, there is also a contribution to neuronal oxidative stress from surrounding activated glia. Activated glia upregulate NADPH oxidase and iNOS expression, releasing superoxide and nitric oxide into the extracellular space. Dopaminergic neuron dysfunction in PD is thought to partly result from glial pro-inflammatory responses [83]. As discussed in Section 3, both un-aggregated and aggregated populations of α-synuclein are capable of activating glia through TLR4 receptors [37]. TLR4 has been shown to mediate microglial clearance of α-synuclein, which may be neuro-protective in some contexts [84]. However, only β-rich oligomers of α-synuclein, and no other α-synuclein species, activate glial TLR2 receptors [85]. This may explain the enhanced pro-inflammatory response of microglia to dopamine-aggregated α-synuclein, relative to un-aggregated α-synuclein [83].

#### 4.2.5. Endoplasmic Reticulum Stress

In the face of misfolded cellular proteins, the endoplasmic reticulum (ER) can mount a protective “unfolded protein response” (UPR) that upregulates the expression of genes that can reduce misfolding, such as molecular chaperones. However, prolonged activation of the unfolded protein response can lead to programmed cell death. Markers of ER stress, such as phosphorylated PERK (protein kinase RNA-like endoplasmic reticulum kinase), are upregulated in PD brain tissue in conjunction with α-synuclein aggregates [86]. Additionally, toxic oligomers of α-synuclein (FILA-1 reactive, proteinase-K resistant) have been found to accumulate in the ER fraction of transgenic A53T α-synuclein mice with age, preceding onset of motor symptoms but increasingly maturing to insoluble S129-phosphorylated aggregates as the disease progresses [87]. Pharmacologically upregulating a UPR pathway, with salubrinal, reduced ER accumulation of FILA-1 reactive α-synuclein oligomers. Salubrinal also reduced Golgi fragmentation and delayed dopaminergic neuron degeneration [88]. There is some evidence that rescuing ER dysfunction could also alleviate the effects of α-synuclein on mitochondria and oxidative stress, so in future this may emerge as an important drug target for PD [89].

Toxic α-synuclein oligomers have been found to more potently activate ER stress, measured by the unconventional splicing of XBP1 (X-box binding protein 1) mRNA, than monomers or fibrils in cultured SH-SY5Y cells. This marker for UPR activation corresponds with the severity of measured reductions in cell viability: 60% for oligomers and 9% for fibrils of α-synuclein. Surprisingly, other amyloidogenic proteins do not all have the same effect on ER stress. Although amyloid-β-42 oligomers and fibrils similarly activated XBP1 splicing in relation to their toxicity, the same relationship did not emerge with toxic oligomers formed from prion protein (PrP106-126) and British dementia amyloid peptide (ABri1-34) [90]. Why α-synuclein oligomers should be special in this regard is not clear, since toxic oligomers of amyloidogenic proteins are thought to share common structures [2].

Potentially, α-synuclein-induced ER stress could stem from the disruption of its normal physiological function by oligomers. ER to Golgi vesicle transport is thought to be regulated by α-synuclein. Overexpression of wildtype α-synuclein impairs ER-Golgi transition, and the A53T disease mutation enhances the severity of this impairment [91]. This can be overcome by overexpressing proteins that promote the ER-Golgi transition, such as Rab1 GTPase, which suppresses α-synuclein toxicity in yeast and mammalian cells [91,92]. At the molecular level, A53T α-synuclein was shown *in vitro* to inhibit the formation of the ER/Golgi SNARE quaternary complexes, which involves the assembly of a 4-helix bundle, important for vesicle docking and fusion. Unexpectedly, no α-synuclein oligomers of any kind were detected during the gel filtration experiment; it only eluted in low molecular weight fractions [91]. The proposed functional effect of α-synuclein on SNARE complex assembly is thought to involve α-synuclein oligomers [33], thus their absence is suspicious and requires further investigation.

#### 4.2.6. Impaired Protein Degradation Systems

Impairment of protein degradation pathways could contribute to the accumulation of α-synuclein aggregates, and α-synuclein oligomers can further impair protein degradation pathways; a reciprocal relationship that has been reviewed in [93]. Effects on both the ubiquitin-proteasome system and autophagy-lysosome pathways have been observed in transgenic α-synuclein models [93]. Reduced proteasome activity has been directly linked to soluble oligomers in α-synuclein-overexpressing PC12 cells, by innovative means. Cell lysates were fractionated with size exclusion chromatography (SEC), and fractions containing active 26S proteasome pooled. The 26S proteasome fractions were found to co-elute with α-synuclein, in complexes larger than 300 kDa. The co-eluting α-synuclein was shown to be oligomeric, running on a native PAGE gel at 150–443 kDa, *i.e.*, oligomers of ~10–30 monomers. Importantly, proteasome activity in these cells could be restored by disrupting of α-synuclein oligomers pharmacologically with Congo Red, which preferentially binds and disturbs β-sheet structure [94]. However, proteasome inhibition may not be exclusive to soluble oligomers of α-synuclein. Previous *in vitro* studies indicated that insoluble fibrils of α-synuclein, as well as β-rich soluble oligomers, inhibited proteasome activity [48,95].

Reduced clearance of specific protein substrates may also arise from the impairment of chaperone-mediated autophagy (CMA) by α-synuclein. Non-aggregated α-synuclein, particularly with A30P or A53T mutations, has the ability to impair the LAMP2A-mediated uptake of CMA substrates into lysosomes [96,97]. The compensatory increase in macroautophagy that follows CMA-blockade may be partly responsible for cell death [96]. It is unclear whether the greater potency of disease-associated mutants on CMA-blockade is due to their monomeric conformation, or their tendency to oligomerize. Similarly when α-synuclein is modified with dopamine at its C-terminal region, it has a striking inhibitory effect on the degradation of CMA substrates *in vitro*. In cells, CMA inhibition of nearly 50% was achieved by treating mixed-neuron (mouse ventral midbrain) cultures with L-DOPA, except when α-synuclein was knocked-out or mutated to prevent dopamine-modification. Again, it is uncertain whether the effect is due to oligomeric species of α-synuclein or the dopamine-modification. However, on isolated lysosomes, dopamine-modified α-synuclein forms organized clusters on the membrane that promote further oligomerization, so oligomers are likely to be involved. Unlike unmodified α-synuclein, dopamine-modified species fail to be translocated into lysosomes by CMA, which could lead to their physical blockade of CMA complexes [98].

## 5. Amyloid Fibrils of α-Synuclein

Although largely discredited since the discovery of toxic α-synuclein oligomers [1], the “amyloid fibril” hypothesis of toxicity has recently re-emerged in publications. Fibrils of α-synuclein form by a nucleated polymerization mechanism. This requires a “seed”, which may be a β-rich oligomer or fragment of amyloid fibril, to which disordered monomers bind and change to an extended β-sheet conformation, causing “elongation” of an amyloid fibril [46]. Amyloid fibrils contain parallel intermolecular contacts between the β-strands of separate component monomers, rather than the anti-parallel intramolecular β-sheet interactions widely seen in soluble oligomers [99]. However, there is evidence for the dynamic inter-conversion of toxic oligomers and fibrils. *In vitro* fibril disassembly, studied by single molecule fluorescence techniques, initially yields a surge in numbers of large proteinase K-resistant oligomers [46]. Cryo-electron microscopy has also documented an apparent transition from annular oligomers to linear elongating protofibrils, during the process of Cu^2+^−dependent aggregation [56]. More stable oligomers may make this transition less easily, and appear to inhibit fibril formation [47].

The role of α-synuclein amyloid fibrils in cell toxicity is a controversial one. Where the toxicity to cells of recombinant oligomers and fibrils has been compared, some studies suggest that fibrils can participate to a greater or lesser extent in mechanisms of toxicity. These mechanisms include membrane permeabilization [47,100], proteasomal impairment [49], and glial activation via TLR2 [85]. However, the precise role that amyloid fibrils play in toxicity is uncertain. Three plausible explanations have been suggested: (a) Mature fibrils are a toxic species in their own right; (b) Toxic oligomers exist in a dynamic equilibrium with fibril assembly/disassembly; (c) The process of active fibril elongation is toxic. The first hypothesis is contradicted by a number of studies mentioned in this review [41,46,76,90,94]. The two other scenarios will be discussed below.

### 5.1. “Toxic Oligomers Exist in a Dynamic Equilibrium with Fibril Assembly/Disassembly”

The most accepted view of the role of amyloid fibrils in cell toxicity is as a passive provider, or destroyer, of toxic oligomers. Fibrils have been shown to disassemble slowly under near-physiological conditions when monomers are removed, and a heterogenous mixture of large and small oligomers and monomers are generated [46]. The products of fibril disassembly, which can be more rapidly induced by sonication, are able to seed growth of new fibrils when monomers are present. This phenomenon appears to occur *in vivo* during the apparent cell-to-cell transmission of α-synuclein aggregates in cell cultures [101] and synucleinopathy models [102]. Pre-formed fibrils applied to primary neurons have been shown to seed growth of endogenous α-synuclein aggregates, in the absence of any endogenous overexpression. Significantly, the α-synuclein inclusions propagated along neuron axons to their cell body, and over time a progressive decline occurred in neuron excitability and connectivity, ending in cell death. These events coincided with depletion of α-synuclein from the presynaptic terminal, and reductions in synaptic proteins involved in vesicle docking, which is strongly suggestive of α-synuclein loss-of-function effects [103]. Furthermore, cells with seeded endogenous α-synuclein aggregates develop defects in autophagic protein clearance [104], and enhanced calcium entry through the plasma membrane [100].

In the β-amyloid field there has been much greater study of the dynamics of fibril formation and toxicity of metastable Aβ42 oligomers. A seminal work by Jan and colleagues illustrates the subtleties of oligomer toxicity, through a series of size-exclusion chromatography (SEC) experiments. Firstly they discovered that SEC of a crude Aβ42 mixture; containing monomers, oligomers, and fibrils; gave purified fractions with greatly attenuated toxicity to primary rat neurons and cell lines. Secondly, through recombining individual fractions it was shown that the key to toxicity was a combination of monomers and high molecular weight “protofibrillar” oligomers. On their own these fractions had no significant toxicity, but when purified monomers and oligomer fractions were combined, enhanced fibril formation and significant toxicity to cells was measured. Interestingly, incubating sonicated fibrils with monomers led to abundant fibril growth but with no significant toxicity. This curious result was expounded by a time-course study of recombinant fibril growth in the medium of cell cultures, and accompanying measurements of cell health. An early decline in mitochondrial health, measured by MTT reduction, was associated with the appearance of elongated protofibrillar oligomers, but far preceded the emergence of mature fibrils. Thus, the toxic oligomer may be a large unstable species formed in the transition from oligomers to elongating fibrils [105].

**Figure 3:**
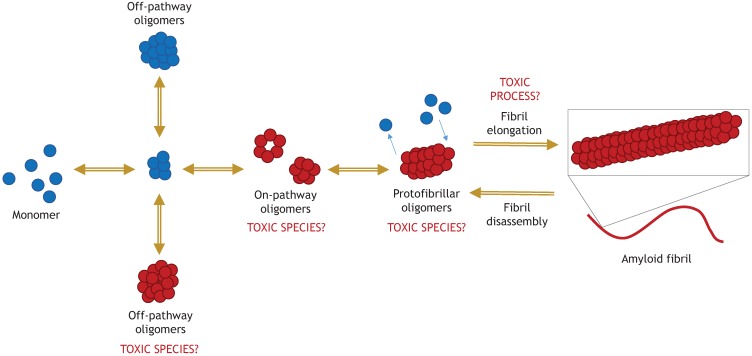
Toxic α-synuclein oligomers in relation to the pathway of amyloid fibril formation. Toxic oligomers have been reported by different studies as being “on-pathway” or “off-pathway” to amyloid fibril formation. Covalent bonding by oxidative modifications may be involved in stabilizing toxic “off-pathway” oligomers. Non-toxic oligomers that are “off-pathway” are stabilized by pharmacological inhibitors of fibril formation, such as baicalein. Toxicity of oligomers is likely to be related to their high levels of β-sheet secondary structure. Additionally, it has been hypothesized that protofibril/fibril elongation may be toxic. Blue circles- Little or no β-structure; Red circles- High β-structure.

No similar study has been performed on α-synuclein, but the potential implication is that toxic oligomers of α-synuclein are “on pathway” to fibril formation. A compelling case for the importance of the fibril-forming pathway was made by enhancing α-synuclein fibrillization *in vivo* using genetic manipulation. Wildtype α-synuclein with a fused CL1 peptide tag was overexpressed in cell culture. The CL1 tag strongly promoted its fibrillization and cellular toxicity, which could be prevented by co-expression of the chaperone HSP70. *In vivo*, the toxicity of aggregating α-synuclein was confirmed by stereotaxic injection of the construct into the substantia nigra of mice. CL1-tagged α-synuclein significantly enhanced degeneration of mouse dopaminergic neurons, compared with untagged α-synuclein, and enhanced formation of LB-like inclusions [106]. However, the idea that toxicity is exclusive to “on pathway oligomers” is not widely supported by studies using recombinant protein, and differs with the methods of oligomer generation. On the one hand, several groups have confirmed the toxicity of “on pathway” oligomers, generated by lyophilization and resuspension, or incubated in the presence of iron/copper [48,54,56]. On the other hand, “off pathway” toxic oligomers were characterized by Lorenzen and co-workers, generated by stirring [47]. Toxic dopamine-modified oligomers also inhibit fibrillization [107]. Clearly, both “on pathway” and “off pathway” α-synuclein oligomers can partake in extracellular toxicity, but it remains to be seen whether “off pathway” α-synuclein oligomers are generated significantly *in vivo*. Figure 3 illustrates the relationship between toxic oligomers and the fibril-forming pathway.

### 5.2. “The Process of Active Fibril Elongation is Toxic”

A slightly more radical hypothesis is that the actual growth of amyloid fibrils, or “elongation”, is a toxic process in protein misfolding diseases. This is speculated to occur through the phenomenon of “lipid extraction” by amyloid fibrils, originally observed in studies of the amyloidogenic “human islet amyloid polypeptide” (IAPP) [108]. Lewy bodies in PD neurons contain a high proportion of lipids [109], and it has been demonstrated *in vitro* that α-synuclein fibrils elongating on the surface of a flat lipid bilayer adsorb membrane lipids. Fluorescently labelled lipids were drawn into growing amyloid fibrils extending out from the plane of the bilayer; an effect accelerated by A53T and E57K α-synuclein variants, but inhibited by stabilizing soluble oligomers with dopamine [110]. Lipid extraction by α-synuclein fibrils has not been studied in cells, but has been shown to disrupt lysosomes and mitochondria isolated from cells [111]. This may potentially explain the perplexing results of studies that varied the proportions of anionic lipids in giant dye-filled vesicles, incubated with either α-synuclein oligomers or fibrils [52,112]. In agreement with previous research, oligomers potently created dye leakage from vesicles where pure anionic phospholipids (e.g., POPG, DOPG, PI) were used [52]. However, the *in vivo* relevance of these membranes is questionable; physiological membranes have a maximum of around 20% anionic lipids. Thus, researchers created a variety of mixed-lipid vesicles with decreasing ratios of anionic to zwitterionic lipids. Vesicles with 50% anionic lipids completely prevented oligomer-induced membrane permeabilization [52]. The surprising subsequent discovery was that α-synuclein fibrils were able to permeabilize vesicles with 20%–100% anionic lipid composition [112]. Thus, amyloid fibrils appear to disrupt membranes in a way that has less dependence on membrane charge, and therefore may not rely on insertion of α-synuclein residues into the hydrophobic core of the membrane, unlike oligomers (see Section 4.2.1). The “lipid extraction” mechanism could adequately explain this, since only a close proximity of fibrils to the membrane would be required. Further investigation is necessary and could be highly enlightening.

## 6. Models for Studying α-Synuclein Toxic Oligomers

Owing to their transient and dynamic nature, studying the toxic effects of α-synuclein oligomers is problematic. The majority of work studying the relative toxicity to cells of α-synuclein has involved recombinant protein that has been aggregated under a variety of laboratory conditions (*i.e*., pH, temperature, agitation, duration), and sometimes with the inducement of metals, organic solvents, or dopamine. Oligomers are a highly heterogenous population in equilibrium with monomers and fibrils, so to improve reproducibility researchers may purify the aggregation reaction by gel filtration, and collect oligomers from the void fraction [113]. However, as noted previously (see Section 4.2), the nature of the oligomers produced can vary dramatically depending on the specific conditions used. Additionally, many studies have used aggregated samples that are not well-characterized. Oligomer populations can be characterized by shape and diameter (electron microscopy, atomic force microscopy, small angle X-ray scattering, dynamic light scattering), or secondary structure composition (circular dichroism, Raman spectroscopy) [113]. Nevertheless, it is a much harder task to characterize their toxicity *in vivo*. The extracellular addition of recombinant oligomers to cell cultures may have some relevance as a model, because the cell-to-cell transmission of α-synuclein aggregates has been shown to involve both exosomal secretion and endocytosis [101,114]. However, by their nature laboratory-produced oligomers are artificial, and may not form naturally *in vivo*. Improved physiological relevance was achieved by one group that isolated oligomers from conditioned media of an α-synuclein transgenic cell line, and applied them to primary rat neurons, resulting in significant cell death. Toxicity could be prevented by immunodepletion of α-synuclein from the conditioned media, or by treatment of the neurons with an oligomer-disrupting chemical called “Congo Red” [114].

The difficulties of studying α-synuclein oligomers within cells has been tackled a number of ways. Size-exclusion chromatography can be used to fractionate cell lysates, for example this has been used to show the co-elution of α-synuclein oligomers with the 26S proteasome [94]. Since conformation appears to be a better marker of oligomer toxicity than size, some researchers have opted to use oligomer-specific conformational antibodies, such as A11 and FILA-1. A11 binds amyloidogenic oligomers of many proteins, but not fibrils or monomers [2], whereas FILA-1 is α-synuclein-specific but binds β-rich oligomers and fibrils [51]. The use of these antibodies aided the discovery that α-synuclein oligomers accumulate in the ER of neurons from aged A53T α-synuclein transgenic mice, and that that this increases with disease progression [87].

Another way that oligomer toxicity has been studied *in vivo* is through overexpression of artificial “oligomer-promoting” mutants of α-synuclein. The idea behind these is that α-synuclein aggregation is enhanced by disease-associated point mutations, but oligomers convert rapidly into fibrils, so the impact of either cannot be distinguished. If α-synuclein mutants were produced by structure-based design that promoted oligomerization but impaired fibril-formation, an augmentation of toxicity relative to fibril-forming mutants would strongly support the “toxic oligomer hypothesis”. This was indeed the case for the artificial E57K α-synuclein mutant. E57K α-synuclein overexpression is more toxic to rat dopaminergic neurons *in vivo* than WT or disease-mutant α-synuclein [115]. E57K α-synuclein is also more prone than WT to forming oligomeric assemblies on the membrane [116], and causes more cytoskeletal disruption in neurites [80]. Other artificial “oligomer-trapping” mutants have proved more controversial. Several proline substitutions in the 31–76 aa region of α-synuclein were shown to inhibit fibril-formation by disrupting β-structure, and appeared to increase α-synuclein neurotoxicity in animal PD models [117]. However, a more recent study with lentiviral-transfected rats showed that only a temporary loss of dopaminergic neurons occurred with artificial pre-fibrillar proline substitution mutants. In contrast wildtype α-synuclein overexpression led to sustained and progressive neurodegeneration [118]. Fibrils may be required for cell-to-cell transmission of α-synuclein, whereas oligomers promote intracellular dysfunction. However, one needs to be cautious in interpreting studies using artificial mutants, because it is difficult to prove that they form a “natural” conformation of α-synuclein oligomers.

Finally, oligomer-disrupting compounds have the potential to be used as tools to probe the *in vivo* effects of toxic oligomers, although so far their employment has been limited. A large number of polyphenolic compounds are known to be potent inhibitors of toxic amyloid oligomers and fibrils [119]. Epigallocatechin gallate (EGCG) is a polyphenol that has been shown to actively remodel α-synuclein monomers, oligomers, and fibrils into forming non-toxic unstructured aggregates [120]. Baicalein is another polyphenol that has been tested on α-synuclein, stabilizing non-toxic oligomers and amorphous aggregates. Applied to inducible α-synuclein PC12 cells, baicalein can prevent specific toxic effects of α-synuclein overexpression, including mitochondrial depolarization and proteasome inhibition [121]. Gallic acid is the simplest anti-amyloid compound: a phenolic acid. Its structure-activity relationship was examined with respect to α-synuclein fibrillization and toxicity, in order to develop a mechanistic model of its effect. Potency was strongly correlated with the number of “OH” groups around a single phenyl ring; gallic acid (3,4,5-trihydroxybenzoic acid) having the maximum number. The planar quinone structure of gallic acid was hypothesized to slot flat into the hydrophobic grooves of β-sheets in toxic oligomers. There it may disrupt the π-π stacking interactions that occur between adjacent aromatic peptides of the protein. Additionally gallic acid appears to bind unstructured oligomers and prevent their conformational change into β-rich oligomers, and potentially prevent further monomer addition to the oligomer [119].

## 7. Conclusions

The disordered nature of the α-synuclein monomer contributes to formation of a diverse range of oligomeric species. One or more of these oligomeric species may contribute to α-synuclein’s ill-defined physiological function, such as modulating SNARE-complex assembly. However, the “wrong” type of oligomer can be highly toxic to cells, for reasons that have not been fully elucidated. The structural features of oligomers that confer toxicity might be related to the high levels of β-sheet secondary structure, the exposure of hydrophobic residues, or the formation of pore structures in membranes. However, no clear or unequivocal link has yet been made between any physical property of α-synuclein “toxic oligomers” and dysfunction in cells. The most studied and well-established property of toxic oligomers is their ability to enhance membrane permeability, although controversy over the mechanism endures. Nevertheless, enhanced membrane permeability by α-synuclein oligomers has not been demonstrated *in vivo*. Perhaps the greatest obstacle is studying α-synuclein oligomers in mechanistic detail within cells, which will require further leaps in technology and methodology. Alternatively, perhaps our fundamental knowledge of the biology of α-synuclein toxicity is still deficient, and we are missing out major pieces of the puzzle. For example, little is known about the impact of toxic oligomers on the functional activities of α-synuclein, such as its involvement in membrane trafficking. A more coherent picture of α-synuclein oligomers and their contribution to synuclein dysfunction and toxicity, will surely be invaluable for future endeavours to develop disease-modifying drugs for synucleinopathy diseases.
